# Association between disability in activities of daily living and phase angle in hemodialysis patients

**DOI:** 10.1186/s12882-023-03400-1

**Published:** 2023-11-29

**Authors:** Junhui Li, Zhi Wang, Qiannan Zhang, Huiping Zhang, Yuxin Shen, Qi Zhang, Guihua Jian, Dongsheng Cheng, Niansong Wang

**Affiliations:** 1 Department of Nephrology, Putuo People’s Hospital, Tongji University School of Medicine, Shanghai, China; 2 Department of Nephrology, Shanghai Sixth People’s Hospital Affiliated to Shanghai Jiao Tong University School of Medicine, 600 Yishan Road, Shanghai, 200233 P.R. China; 3 Tian Lin Community Health Center, Xuhui District, Shanghai, China

**Keywords:** Activities of daily living, Phase angle, Hemodialysis, Disability

## Abstract

**Background:**

Disability in activities of daily living (ADL) significantly increases the risk of mortality among patients undergoing hemodialysis. Malnutrition and decreased exercise capacity are closely correlated with ADL disability. Phase angle (PhA) has been proposed as a measure of nutritional status and exercise capacity. This study aims to investigate the prevalence of ADL disability in hemodialysis patients and its association with PhA.

**Methods:**

A prospective, observational study was conducted, involving hemodialysis patients treated between November 2019 and January 2020 in an affiliated hospital of Chinese university. ADL was measured using both basic ADL (BADL) scales and instrumental ADL (IADL) scales. PhA measurements were obtained using a BIA device while the patients were in the supine position after dialysis.

**Results:**

A total of 237 hemodialysis patients with a mean age of 60.01 ± 13.55 years were included in this study. The prevalence of disability in ADL was 43.5%. Multivariable analysis results showed a robust association between low PhA and disability in both BADL and IADL (for each unit decrease in PhA: odds ratio 4.83 [95% CI: 2.56–9.0], and 3.57 [95% CI: 2.14–5.95], respectively). The optimal cut-off values of PhA for disability in BADL and IADL were 4.8 and 5.4, with the area under the ROC curve (AUC) were 0.783 (0.727, 0.835) and 0.799 (0.743, 0.848), respectively.

**Conclusions:**

Low PhA is strongly associated with disability in ADL in hemodialysis patients. These findings suggest that PhA may serve as a potentially objective measure of ADL disability in hemodialysis patients.

## Background

Due to the protein-energy malnutrition and decreased exercise capacities, hemodialysis patients often experienced reduced physical mobility and a decline in independent living ability. This not only leads to a decrease in quality of life but also results in a significant increase in healthcare costs [1, 2]. ADL is a common index to evaluate capacity of individuals to perform the activities and tasks, and the presence of ADL disability significantly increases the risk of mortality among hemodialysis patients [3]. Therefore, early recognition of disability in ADL and subsequent improved care are crucial for enhancing the outcomes of patients on hemodialysis.

The phase angle (PhA) is an index derived from bioelectrical impedance analysis (BIA) to evaluate nutritional status [4]. PhA is calculated from BIA using the following two measures: reactance (cell membrane-specific resistance, *Xc*) and resistance (intracellular and extracellular resistance, *R*). Because PhA is directly derived from the fixed formula using the original data *R* and *Xc*, it is less affected by the fluid distribution. Studies have indicated that PhA could show the health and nutrition status of cells [4, 5]. There is a positive correlation between PhA and cell membranes integrity and function. PhA is widely recognized as an indicator of nutritional status, exercise capacity, disease severity, and disease prognosis [6–8]. Malnutrition and decreased exercise capacity are both closely related to disability in ADL. Hence, our hypothesis was that low PhA could be associated with disability in ADL in dialysis patients.

Therefore, this study was performed to investigate the prevalence of disability in ADL and the association between PhA and disability in ADL in patients on hemodialysis.

## Methods and materials

### Subjects and data acquisition

**Fig. 1:**
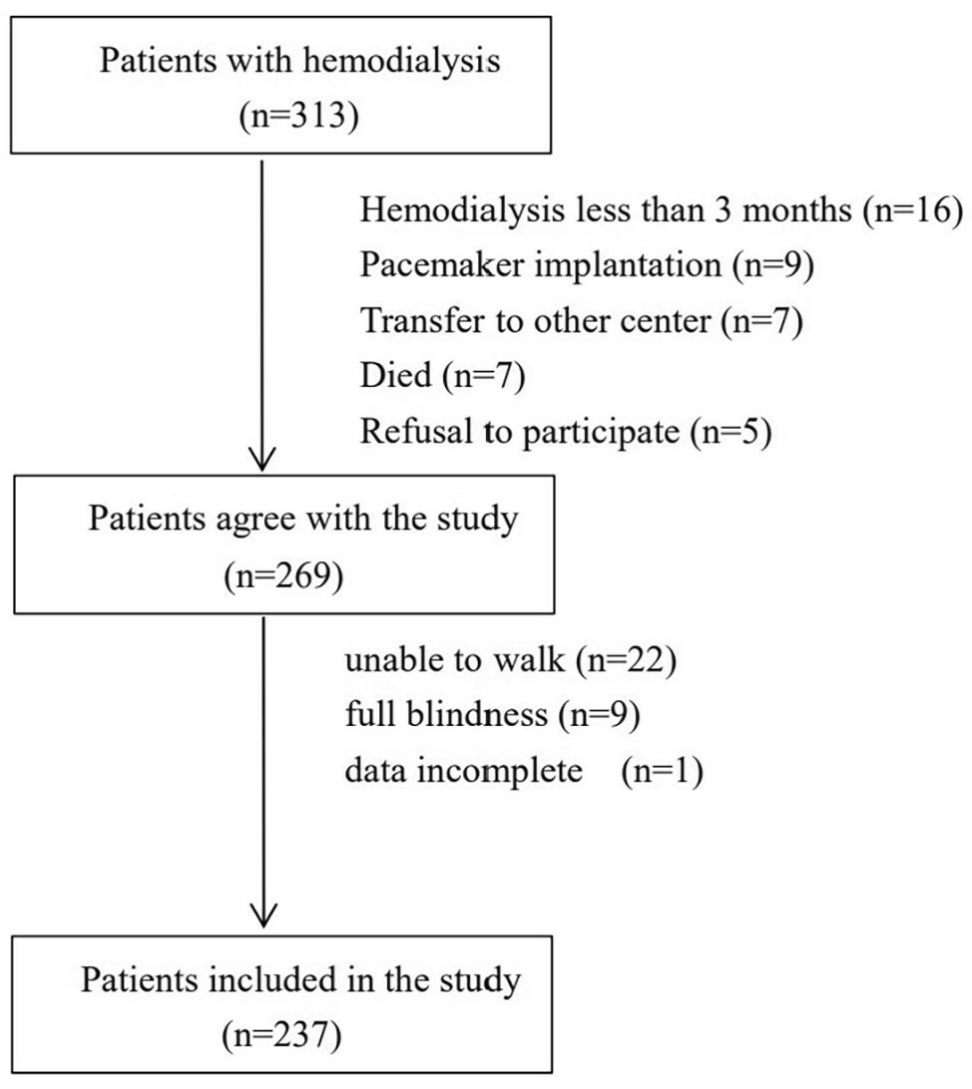
The patient flowchart

We conducted a prospective observational study from November 2019 to January 2020 at an affiliated hospital of Chinese university. The inclusion criteria were hemodialysis patients who had been receiving treatment for a minimum of 3 months and were aged 18 years or older. The exclusion criteria were applied as follows: major individuals with limb loss, unable to walk, full blindness, and pacemaker implantation. The patients with implanted cardiac electronic devices were not enrolled to the study due to the inability to perform BIA measurements in accordance with the manufacturer’s recommendations. As shown in Fig. 1, a total of 313 patients were initially screened in the study, but 76 subjects were subsequently excluded (hemodialysis lasted less than 3 months [*n* = 16]; pacemaker implantation [*n* = 9]; transfer to other center [*n* = 7]; died [*n* = 7]; unable to walk [*n* = 22]; full blindness [*n* = 9]; refusal to participate [*n* = 5]; data incomplete [*n* = 1]). Ultimately, 237 subjects were enrolled, which was approved by the Ethics Committee of Hospital (SH-2018-125). All subjects signed written consent forms, which included agreeing to the collection of their demographic information, lifestyle behaviors (smoking and drinking habits), medical history, dialysis vintage, laboratory data (hemoglobin and serum albumin within one month prior to the start of the study) and to complete the required tests. Smoking and drinking habits were categorized as “never,” “former,” or “current”.

Body mass index (BMI) was calculated based on dry weight and in kilograms divided by height in meters squared. The whole body 50 kHz PhA was measured using a BIA device (InBody S10, Seoul, Korea). To avoid the impact of excessive water retention on BIA results, PhA was measured in the supine position right after the second or third dialysis during one week [9].

In this study, we utilized the Chinese version of ADL scale, which is widely recognized and employed [10–13]. Functional ability is the actual or potential capacity of an individual to perform the activities and tasks that can be normally expected. The 14-item ADL scale, including the 6-item basic ADL (BADL) and 8-item instrumental ADL (IADL) scales, were conducted to evaluate each patient’s disability [14]. The BADL scale included six items: bathing, eating, dressing, indoor activities, using the toilet, and control of urination and bowel movement. The evaluation results of each item were divided into 3 categories: requiring no assistance, requiring partial assistance, and requiring full assistance. Assistance of any of the aforementioned items (including partial and full assistance) was defined as impaired BADL; otherwise, it was defined as intact BADL. The IADL scale included eight items: shopping, outdoor activities, food preparation, housekeeping, laundry ability to use telephone, taking medication, and ability to handle finances. The evaluation results of each item were divided into three categories: requiring no assistance, requiring partial assistance, and requiring full assistance. Assistance of any of the aforementioned items (including partial and full assistance) was defined as impaired IADL; otherwise, it was defined as intact I ADL. These scales have been proven by numerous studies to be valid for evaluating disability of patients with hemodialysis [9, 15, 16].

### Statistical analysis

Categorical data were presented as numbers (percentage). Continuous data were assessed for normality using a Shapiro-Wilk test. Continuous data were expressed as mean ± standard deviation for normally distributed variables, and as median (interquartile range) for non-normally distributed variables. For comparison of continuous variables between two groups, the unpaired Student’s t-test (normal distribution) and Mann-Whitney U-test (nonnormal distribution) were used. The chi-squared test was used for categorical variables. Univariable and multivariable logistic regression analysis were used to determine the association of disability with PhA. Multivariable analysis adjusted for age, sex, history of hypertension and diabetes mellitus, BMI, dialysis vintage, smoking and drinking habits, hemoglobin, and serum albumin level. Multi-collinearity was checked by variance inflation factors and model fitness was assessed by Hosmer Lemeshow test. In the present study, we drew the receiver operating characteristic (ROC) curves corresponding to disability in BADL and IADL with PhA. The areas under the ROC curves (i.e., AUCs) were calculated. The point of the ROC curve closest to the upper left corner was used as the cut-off value of PhA to calculate the corresponding sensitivity, specificity, positive predictive value, and negative predictive value. All statistical analysis was performed using MedCalc software version 18.2.1 (MedCalc Software, Ltd., Ostend, Belgium). *P* < 0.05 was considered as a statistically significant difference.

## Results

### General characteristics

A total of 237 hemodialysis patients were included in the study,with an average age was 60.01 ± 13.15 years. Patients were predominantly male (67.5%), the prevalence of hypertension was 83.5%, and the dialysis vintage was 2.56 (1.14, 4.85) years. The prevalence of disability in BADL was 21.1% and that of disability in IADL was 41.4%. Demography data was as described in our previous published article (as shown in Table 1) [9]. As shown in Table 1, in comparison to the group with independence in BADL, the group with dependence in BADL was older (*P* = 0.002), and had lower hemoglobin (*P* = 0.020) as well as lower serum albumin level (*P* < 0.001). Compared with IADL independence group, IADL dependence group was older (*P* < 0.001), and had lower serum albumin level (*P* = 0.003).

**Table 1:** Participants’ Characteristics According to the BADL and IADL dependence

Variables	BADL-0 (n = 187)	BADL-1 (n = 50)	P value	IADL-0 (n = 139)	IADL-1 (n = 98)	*P*-value
Phase angle	5.41 ± 0.97	4.38 ± 0.92	< 0.001	5.62 ± 0.90	4.58 ± 0.92	< 0.001
Age, yr	59.56 ± 12.52	66.46 ± 14.11	0.002	56.35 ± 12.36	67.62 ± 11.30	< 0.001
Female, *n (*%)	63 (33.7)	14 (28.0)	0.445	43 (30.9)	34 (34.7)	0.543
BMI, kg/m^2^	22.68 ± 3.71	21.97 ± 3.37	0.202	22.84 ± 3.76	22.09 ± 3.45	0.111
Hypertension, *n* (%)	153 (81.8)	45 (90.0)	0.166	112 (80.6)	86 (87.8)	0.142
Diabetes, *n* (%)	74 (39.6)	23 (46.0)	0.411	52 (37.4)	45 (45.9)	0.190
Dialysis vintage, yr	2.62 (1.21–5.05)	1.92 (1.04–4.48)	0.140	2.65 (1.12–5.04)	2.31 (1.23–4.58)	0.546
Smoking, *n* (%)			0.225			0.018
Never	91 (48.7)	18 (36.0)		65 (46.8)	44 (44.9)	
Former	54 (28.9)	20 (40)		35 (25.2)	39 (39.8)	
Current	42 (22.5)	12 (24)		39 (28.1)	15 (15.3)	
Drinking, n (%)			0.401			0.761
Never	94 (50.3)	24 (48.0)		72 (51.8)	46 (46.9)	
Former	67 (35.8)	22 (44.0)		50 (36.0)	39 (39,8)	
Current	26 (13.9)	4 (8.0)		17 (12.2)	13 (13.3)	
Hemoglobin, g/L	110.7 ± 15.8	104.6 ± 18.7	0.020	110.1 ± 15.8	108.4 ± 17.8	0.433
Serum Albumin, g/L	41.8 ± 3.7	38.7 ± 5.4	< 0.001	41.8 ± 3.6	40.1 ± 5.1	0.003
BADL score	12.0(12.0–12.0)	11.0(9.0–11.0)	< 0.001	12.0(6.0–14.0)	12.00 (11.0–12.0)	< 0.001
IADL score	16.0 (15.0–16.0)	8.0 (5.0–13.0)	< 0.001	16.0 (16.0–16.0)	12.0 (6.0–14.0)	< 0.001

Values are presented as mean (SD) for normally distributed continuous values, median (interquartile range) for skewed continuous values, and N (%) for categorical values. BMI, body mass index; BADL, basic activities of daily living; IADL, instrumental activities of daily living

The BADL-1 and LADL-1 mean that of disability in ADL, and the BADL-0 and LADL-0 mean that of non-disability in ADL

### Analysis of the association between disability in ADL and PhA

As shown in Table 2, the univariable logistic model analysis showed that, in comparison to individuals in the third tertile for PhA (the highest numerical value group), the odds ratio (OR) for disability in BADL and IADL were greater than ten in the first tertile (*P* < 0.001). According to the multivariable analysis, which incorporated adjustments for age, sex, history of hypertension and diabetes mellitus, BMI, dialysis vintage, smoking and drinking habits, hemoglobin, and serum albumin level, compared with the third tertile, patients from the first tertile had an adjusted OR of 18.50 for disability in BADL (95% CI: 3.89–88.05) and 17.37 for disability in IADL (95% CI: 5.19–58.08), respectively. A similar result was observed with PhA for every unit decrease. Multivariable logistic regression analysis demonstrated that low PhA was strongly associated with disability in BADL and IADL (per unit decrease of PhA: OR 4.83 [95% CI: 2.56–9.0], and 3.57 [95% CI: 2.14–5.95], respectively).

**Table 2:** Univariable and multivariable logistics regression for the association of disability with tertiles of phase angle

Phase angle	Disability in BADL		Disability in IADL	
	Univariable OR	Multivariable OR*	Univariable OR	Multivariable OR*
T3	1.00 (Reference)	1.00 (Reference)	1.00 (Reference)	1.00 (Reference)
T2	4.86 (1.35, 17.52), *P* = 0.016	4.91 (1.17, 20.58), *P* = 0.029	5.23 (2.23, 12.27), *P* = 0.001	4.00 (1.45, 10.98), *P* = 0.020
T1	15.66 (4.53, 54.11), *P* < 0.001	18.50 (3.89, 88.05), *P* < 0.001	19.48 (8.07, 47.00), *P* < 0.001	17.37 (5.19, 58.08), *P* < 0.001

*Adjusted for age, sex, history of hypertension and diabetes mellitus, BMI, dialysis vintage, smoking and drinking habits, hemoglobin, and serum albumin level. BADL, basic activities of daily living; IADL, instrumental activities of daily living; OR, odds ratio. Values within parentheses are the 95% confidential intervals

### The ROC curves of phase angle in the whole study population

**Fig. 2:**
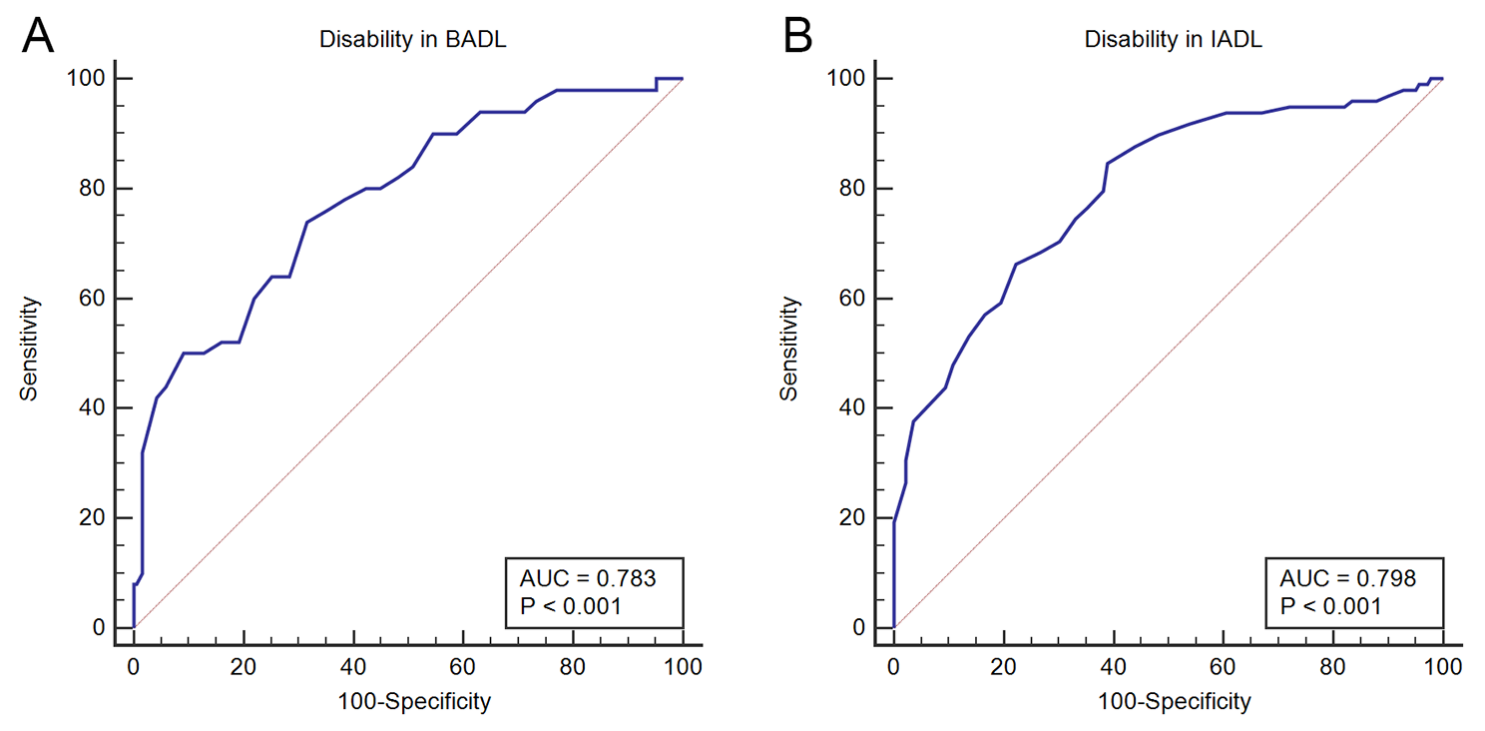
The receiver operating curves of phase angle in the whole study population. (**A**) Disability in BADL. (**B**) Disability in IADL. BADL, basic activities of daily living; IADL, instrumental activities of daily living

As shown in Fig. 2; Table 3, the AUCs for disability in BADL and IADL were 0.783 (0.727, 0.835) and 0.799 (0.743, 0.848), respectively. In addition, the sensitivity, specificity, positive likelihood ratio, and negative likelihood ratio are shown in Table 3. The optimal cut-off values of PhA for BADL and IADL were 4.8 and 5.4, respectively. The respective sensitivities in screening for disability in BADL and IADL were 74.0% and 84.7%, and the respective specificities were 68.5% and 61.2%, respectively.

**Table 3:** Sensitivity/specificity analyses and receiver operating curve models for phase angle validation

Variables	Cutoff value	Sensitivity (%)	Specificity (%)	LR+	LR-	AUC
Disability in BADL						
Total	PhA ≤ 4.8	74.0	68.5	2.35	0.38	0.783 (0.727, 0.835)
Men	PhA ≤ 5.5	86.1	50.0	1.72	0.28	0.747 (0.673, 0.813)
Women	PhA ≤ 4.1	85.7	85.7	6.00	0.17	0.911 (0.824, 0.964)
Disability in IADL						
Total	PhA ≤ 5.4	84.7	61.2	2.18	0.25	0.799 (0.743, 0.848)
Men	PhA ≤ 5.4	79.7	64.6	2.25	0.31	0.760 (0.687, 0.824)
Women	PhA ≤ 4.5	73.5	88.4	6.32	0.30	0.874 (0.779, 0.939)

LR+, positive likelihood ratio; LR-, negative likelihood ratio; AUC, area of receiver operating curve; BADL, basic activities of daily living; IADL, instrumental activities of daily living. Values within parentheses are the 95% confidential intervals

### Comparison of phase angle in men and women

As shown in Table 3, males and females displayed different cut-off values for PhA. The optimal cut-off values of PhA in BADL in males and females were 5.5 and 4.1, respectively. The optimal cut-off values of PhA in IADL in males and females were 5.4 and 4.5, respectively. The AUCs for disability in BADL and IADL in males were.

0.747 (0.673, 0.813) and 0.760 (0.687, 0.824), and those in females were 0.911 (0.824, 0.964) and 0.874 (0.779, 0.939).

## Discussion

In the present study, we investigated the prevalence of disability in ADL and, for the first time, reported its association with PhA in patients on hemodialysis. The findings of our study showed that disability in ADL is common issue in hemodialysis patients and low PhA is significantly associated with disability in ADL in hemodialysis patients.

As a result of protein-energy malnutrition and decreased exercise capacity, hemodialysis patients often suffer from the loss of mobility, even to the point of disability, which leads to the inability to live independently [17, 18]. A small cross-sectional study of hemodialysis patients indicated that more than 80% of patients exhibited IADL disability, and the prevalence of BADL disability exceeded 40% [19]. Consistently, another study showed that nearly 30% of hemodialysis patients had disabilities in ADL and more than 60% had disabilities in IADL [20]. It was reported that patients with disability in ADL had a higher incidence and severity of post-dialysis fatigue [20]. Moreover, several studies have reported that disability in ADL was a strong predictor of mortality in hemodialysis patients [21–23]. In our study, we found that the prevalence of disability in BADL was 21.1%, that of disability in IADL was 41.4%, and that of disability in ADL was 43.5% of the hemodialysis patients in our cohort. Therefore, disability is common in hemodialysis patients.

PhA has been investigated as a prognostic marker for mortality in various diseases, including cancer, cardiac diseases, kidney diseases, and others [24–26]. Previous studies have shown that PhA as a marker reflecting the nutritional status of chronic kidney disease patients both with maintenance dialysis and without [27–29]. Notably, malnutrition among hemodialysis patients often co-exists with chronic inflammation. A previous study found that longitudinal changes in bioimpedance PhA reflect inverse changes in serum IL-6 levels in maintenance hemodialysis patients [30]. It was found that longitudinal changes in PhA appear to be reliable in detecting changes in nutritional and inflammatory parameters over time, that may contribute to the understanding of its prognostic utility. Low-grade systemic inflammation is also associated with functional disability in elderly people affected by dementia [31]. Additionally, PhA has been associated with muscle mass, strength, physical performance, quality of life or mortality [32–35]. In our study, after adjusting for multiple variables, logistic regression analysis confirmed that low PhA remained strongly associated with disability in hemodialysis patients, further expanding our understanding of the clinical value of PhA. Consistent with previous studies [36–38], our findings demonstrated that male subjects had higher PhA values. The sex difference in PhA might be associated with higher proportion of muscle and water in males, whereas female tend to have a higher proportion of body fat in females. However, due to the limited sample size of our study, further research is needed to validate this sex related disparities in PhA and ADL disability among hemodialysis patients.

There are several limitations in the study. First, as this was a single-center study, the generalizability of the study results might be limited. Second, this study adopted a prospective, observational design, so the cause-effect association was not solid enough. Third, there are many comorbidities in hemodialysis, such as hypertension, diabetes and coronary heart disease, but our study only included hypertension and diabetes, omitting other comorbidities and inflammatory indicators that could potentially influence the results. Lastly, the sample size in our study was relatively small, and this may have masked other possible interactions.

In conclusion, our study shows disability in ADL is common in hemodialysis patients. Furthermore, PhA has the potential to serve as an objective measure of disability in hemodialysis patients. In the future, further research could explore the association between low PhA and new-onset disability in ADL, and clarify whether changes of PhA can be used to evaluating functional improvements or declines in hemodialysis patients.

## Data Availability

The datasets used and/or analyzed during the current study are available from the corresponding author on reasonable request.
